# Complete Genome Sequence of Original Material Used To Derive the WHO International Standard for Human Polyomavirus BK DNA

**DOI:** 10.1128/MRA.00911-19

**Published:** 2019-10-24

**Authors:** Adrian Jenkins, Sheila Govind, Clare Morris, Neil Berry

**Affiliations:** a Division of Infectious Disease Diagnostics, NIBSC, South Mimms, United Kingdom; DOE Joint Genome Institute

## Abstract

The complete genomic sequence was determined for the original biological material used to derive the WHO international standard for BK polyomavirus (BKV) DNA. The entire coding sequence and noncoding regions were assigned BKV subtype 1, subgroup 1b-1. This information will aid development and evaluation of human BKV DNA amplification assays.

## ANNOUNCEMENT

Human polyomavirus BK (BKV) is a member of the genus *Orthopolyomaviridae*, belonging to the family *Polyomaviridae*. It represents an important human pathogen in immunocompromised hosts, especially in cases of graft loss following renal transplantation, and has been causally linked with BKV nephropathy. BKV is a small DNA virus first identified in 1971 (1), existing as an ∼5.1-kb double-stranded circular genome coding for different functional regions, including early antigens (denoted T and t), and four late capsid genes, including coding regions for capsid proteins VP1 to VP3. Noncoding regions contain transcriptionally relevant promoter/enhancer elements. Screening of biological specimens for BKV DNA using molecular techniques represents a key part of virological diagnostic procedures for immunosuppressed individuals, especially renal transplant patients. Here, we determine the genome of an early BKV isolate subsequently used to generate the first international standard (IS) for BKV DNA.

Biological reference materials generated at the National Institute for Biological Standards and Control (NIBSC) were derived from an original isolate of BKV donated by the Gardner laboratory, as previously described (2). The donated material was used to generate the baseline sequence reported here to provide a means of making subsequent comparisons of sequence heterogeneity of BKV isolates and IS derivatives. Briefly, genomic DNA was extracted from BKV-infected cells and supernatant using QIAamp viral minikits according to the manufacturer’s instructions (Qiagen, Germany) in conjunction with QIAcube instrumentation (Qiagen). Extracted DNA was subjected to virus-specific PCR amplification with six overlapping amplicons encompassing the entire BKV genome, as shown in Table 1. Amplicons were directly sequenced with combinations of oligonucleotides in forward (F) and reverse (R) directions, as indicated.

**TABLE 1:** Nucleotide positions referenced according to the Dunlop strain (GenBank accession number V01108)^a^

Amplicon	Sequence name (orientation)	Primer sequence	Nucleotide position
1	BKPP1 (F)	AGGCCTCAGAAAAAGCCTCC	49–68
	SeqB11 (R)	CCTCCTGTGAAAGTCCCAAAATACC	2246–2222
2	SeqB10 (F)	CTAAAAACCCAACAGCCCAGTCC	2102–2124
	SeqB21 (R)	TGCCTTAACTAGAGATCCATAC	4102–4081
3	SeqB10 (F)	CTAAAAACCCAACAGCCCAGTCC	2102–2124
	SeqB23 (R)	ATTCTCAACACTCAACACCACCC	4448–4426
4	SeqB18 (F)	CCACCACACAAATCTAATAACC	3470–3491
	SeqB26 (R)	AATGGAGCAGGATGTAAAGGTAGC	4971–4948
5	SeqB22 (F)	TATACACAGCAAAGCAGGCAAG	4317–4338
	SeqB11 (R)	CCTCCTGTGAAAGTCCCAAAATACC	2246–2222
6	SeqB22 (F)	TATACACAGCAAAGCAGGCAAG	4317–4338
	SeqB15 (R)	AATCACAATGCTCTTCCCAAGTC	2861–2839

a Underlines indicate previously described sequences (7).

Each of the six overlapping fragments, ranging from ∼1,500 to ∼3,500 bp long, was sequenced directly using an ABI PRISM BigDye Terminator kit with a thermoprofile of 25 cycles (96°C, 30 s; 50°C, 15 s; 60°C, 4 min) on a 3130XL sequencer (Applied Biosystems Ltd.) in forward and reverse directions. Sequences were assembled and analyzed using Geneious v. 10.2 software, with percentage similarities determined at the nucleotide level using the Geneious pairwise alignment tool with default parameters. A contiguous sequence represented by 5,141 nucleotides (nt) contained a total GC content of 39.3%, with ∼98% nucleotide sequence homology to the isolate LAB-22 (GenBank accession number AB301093) previously reported (3).

The complete genome sequence reported here represents the original starting material from which the first WHO BKV DNA IS was generated. Phylogenetic and sequence identity analyses indicate a sequence most closely aligned with BKV subtype 1, subgroup 1b-1 strains, in accordance with the subtyping/subgrouping classification of BKV sequences based on nucleotide differences in the VP1 region to classify BKV genotypes (4).

Consensus Sanger sequencing-derived information from early isolates of this kind provides a bridge to information generated with more highly passaged virus stocks, where inconsistencies in sequence composition and target identity have been revealed using deep sequencing technologies (5). This information will support genome amplification assay development for BKV DNA detection and quantification in environmental samples in a clinical setting, assisting in the harmonization and commutability assessment of diagnostic assays for human polyomavirus BK (2, 6).

### Data availability.

The genome sequence reported here, from which the first WHO IS for BKV DNA was derived, is filed in GenBank under accession number MN135851. The Dunlop strain is listed under GenBank accession number V01108, and the complete genome sequence of the BK polyomavirus DNA (LAB-22 isolate) (3) is listed under GenBank accession number AB301093.
